# Associations of siesta and total sleep duration with hypertension or cardiovascular diseases in middle‐aged and older adults

**DOI:** 10.1002/clc.23954

**Published:** 2022-12-12

**Authors:** Ling Lin, Jingyi Huang, Zixuan Liu, Pengfei Chen, Canyan Huang

**Affiliations:** ^1^ Department of Emergency Nursing Fujian Medical University Union Hospital Fuzhou China

**Keywords:** cardiovascular disease, hypertension, siesta, sleep duration

## Abstract

**Background:**

Whether the ratio of siesta duration in the total sleep duration was associated with the occurrence of hypertension or cardiovascular diseases (CVDs) was unclear.

**Hypothesis:**

To explore the associations of siesta, and siesta ratio with hypertension or CVDs in middle‐aged and older adults.

**Methods:**

This cohort study collected the data of 9247 middle‐aged and older adults. The associations of siesta, and siesta ratio with hypertension were analyzed in 7619 participants while the associations of siesta, and siesta ratio with CVDs were analyzed in 8685 participants via univariate and multivariate logistic regression analysis.

**Results:**

Total sleep duration < 6 h (odd ratio [OR] = 1.168, 95% confidence interval [CI]: 1.023−1.335) and siesta ratios ≥ 0.4 (OR = 1.712, 95% CI: 1.129–2.594) were associated with increased risk of hypertension in middle‐aged and older adults. Siesta ratio ≥ 0.4 was linked with higher risk of hypertension in males aged ≥ 60 years and total sleep duration < 6 h was correlated with elevated risk of hypertension in males < 60 years. The risk of CVDs was elevated in people with siesta duration < 0.5 h (OR = 2.053, 95% CI: 1.323−3.185). In females ≥60 years, the sleep duration at night < 6 h was associated with increased risk of CVDs. In females < 60 years, increased risk was observed in those with siesta duration < 0.5 h and sleep duration at night < 6 h.

**Conclusion:**

Short sleep duration or high siesta ratio were associated with higher risk of hypertension. Short siesta duration, sleep duration at night or total sleep duration were correlated with an elevated risk of CVDs.

## 1 INTRODUCTION

Hypertension is a prevalent chronic disease influencing nearly 1 billion adults and associated with over 10.8 million deaths every year.^1^ Hypertension has caused a heavy burden of cardiovascular morbidity and mortality, which leads to an estimated cost of $48.6 billion annually.^2^ High blood pressure affects multiple organs and hypertension has been identified to be a primary modifiable risk factor for cardiovascular diseases (CVDs), such as coronary heart disease, myocardial infarction, and stroke.^3^, ^4^ CVDs remains to be the major cause of mortality, morbidity and disability.^5^ In the past three decades, the global prevalence of CVDs had an increase from 271 million in 1990 to 523 million in 2019, and the CVD mortality has risen from 12.1 million in 1990 to 18.6 million in 2019, which represents about one‐third of the annual deaths all over the world.^6^ Although great advances have achieved in prevention, early diagnosis and treatment, hypertension and CVDs still bring considerable socioeconomic burden to the health systems and community. More effective health care planning and prevention of hypertension and CVDs are essentially needed.

Currently, some research proposed that lifestyle changes may have significant effects on blood pressure control and further influence the occurrence of CVDs.^7^ Sleep status is associated with autonomic nervous system function and other physiologic events, which further affects blood pressure in people.^8^ Unhealthy sleep status was reported to change the blood pressure response and increase the risk of hypertension.^9^ Bertisch et al. found that short sleep duration was linked with the risk of incident CVDs and all‐cause mortality in those patients.^10^ Sleep includes the daytime sleep and nighttime sleep. As a kind of daytime sleep, siesta is a prevalent lifestyle in many countries including China, and according to the data from Chinese Center for Disease Control and Prevention, 39.3% of Chinese adults aged 15−69 years take siesta regularly, and that the rate of siesta was the highest among people aged 60−69 years.^11^, ^12^ Previously, various studies explored the associations between siesta and hypertension or CVDs and revealed that siesta was associated with decreased risk of hypertension or CVDs and other studies also found that too long siesta was associated with increased risk of hypertension.^12^, ^13^, ^14^ Too long siesta may decrease the sleep duration at night and affect the total sleep duration of people, indicating that the percentage of siesta in the total sleep duration may be associated with the risk of hypertension or CVDs.^15^ Whether the ratio of siesta duration in the total sleep duration was associated with the occurrence of hypertension or CVDs was still unclear.

In the current study, we analyzed the associations between siesta, sleep duration at night, total sleep duration and the percentage of siesta in total sleep duration and the incidence of hypertension or CVDs in the middle‐aged and older adults based on the data from the China Health and Retirement Longitudinal Study (CHARLS). We also stratified the analysis by age and sex as age and gender were reported to be associated with the risk of hypertension in previous study.^16^ The findings of this study might provide a reference for plan the sleep time and duration of middle‐aged and older adults for the prevention of hypertension and CVDs.

## 2 METHODS

### 2.1 Study design and population

This cohort study collected the data of 10 113 middle‐aged and older adults without hypertension or CVDs and with sleep data from CHARLS database. CHARLS is a nationally representative longitudinal survey conducted between June 2011 and March 2012. The survey investigated the social, economic, and health circumstances of 17 708 community‐residents ≥ 45 years old. A total of 150 county‐level units were randomly selected from the 28 provinces and autonomous regions in mainland China using the probability‐proportional‐to‐size sampling technique. The respondents in CHARLS are followed every 2 years via a face‐to‐face computer‐assisted personal interview. Physical measurements are made at every 2‐year follow‐up, and blood sample is collected once in every two follow‐up periods.^17^ In our study, participants missed the data on hypertension or CVDs during follow‐up were excluded. Finally, 7604 participants were analyzed to evaluate the associations between siesta, sleep duration at night, total sleep duration and siesta ratio and hypertension, and people were divided into the hypertension group (*n* = 2075) and nonhypertension group (*n* = 5529). Eight thousand six hundred seventy people were analyzed to measure the associations between siesta, sleep duration at night, total sleep duration and siesta ratio and CVDs, and participants were divided into the CVD group (*n* = 986) and non‐CVD group (*n* = 7684). The study was conducted in accordance with the Declaration of Helsinki, and the original CHARLS was approved by the Ethical Review Committee of Peking University, and all participants signed informed consent at the time of participation.

### 2.2 Covariables

The covariables included in this study were age (<60 years or ≥60 years), gender, marriage (married, widowed, or others), registered permanent residence (Agricultural residence, Nonagricultural residence, and Unified Residence or do not have registered permanent residence), education (no formal education illiterate, did not finish primary school but capable of reading or writing, elementary school, middle school/high school/vocational school, Two/Three‐Year College/Associate degree/Four‐Year College/Bachelor's degree/Post‐graduate, or Master's degree), smoking (yes, quit smoke, no, or unknown), drinking (yes [drink more than once a month], yes [drink less than once a month], no or unknown), vigorous activity (yes or no), moderate activity (yes or no), walk activity (yes or no), dyslipidemia (yes or no), diabetes mellitus dysglycemia (yes or no), the 10‐item Center for Epidemiological Studies Depression Scale (CES‐D‐10) (yes or no), body mass index (BMI) (<18.5 kg/m^2^, 18.5 kg/m^2^ −23.9 kg/m^2^ or ≥24 kg/m^2^).

### 2.3 Independent variables and definition

Independent variables were analyzed including the siesta (yes or no), siesta duration (0 h, 0 h < duration < 0.5 h, 0.5 h ≤ duration < 1 h, 1 h ≤ duration < 1.5 h or ≥1.5 h), sleep duration at night (<6 h, 6 h ≤ duration < 8 h or ≥8 h), total sleep duration (<6 h, 6 h ≤ duration <8 h or ≥8 h), and siesta ratio (0 h, 0 h < ratio < 0.2 h, 0.2 h ≤ ratio < 0.4 h or ≥.4). Self‐reported sleep duration was obtained via a structured questionnaire according to the question “During the past month, how many hours of actual sleep did you get at night (average hours for one night)?” and question “During the past month, how long did you take a nap after lunch?” Total sleep duration indicated the total hour the participants actually slept every day. Afternoon nap duration was the total hour the participants slept after lunch every day. Afternoon nap ratio referred to afternoon nap duration to total sleep duration.

### 2.4 Outcome variables, definitions, and follow‐up

Two outcomes were evaluated in our study including the occurrence of hypertension in people and the occurrence of CVDs in participants. All participants were followed up for 3 years via questionnaires. The follow‐up was discontinued when an outcome event was observed and the follow‐up of all participants was ended in 2018.

The median follow‐up was 6.51 ± 1.11 years.

Hypertension was confirmed by the patient's self‐report of a physician's diagnosis or in combination with health assessment and medication data in CHARLS survey when satisfied any of the four criteria: systolic blood pressure (SBP) ≥ 140 mmHg, diastolic blood pressure (DBP) ≥ 90 mmHg, self‐report of a diagnosis of hypertension, or currently taking antihypertensive medication. DBP and SBP levels were measured three times with at least 45‐s intervals using a digital sphygmomanometer (Omron TM HEM‐7200 Monitor, Co., LTD.).

At baseline and follow‐up, participants were asked whether they had been diagnosed by a doctor to have stroke or cardiac events (including heart attack, coronary heart disease, angina, and congestive heart failure or other heart problems). A positive answer to the question was defined as having CVDs.

### 2.5 Statistical analysis

The enumeration data were shown as *n* (%). *χ* ^2^ test or Fisher's exact probability method was used for comparison between groups. Missing data on age and BMI were dealt by multiple imputation during logistic regression. Univariate and multivariate logistic regression model were applied for exploring the associations of siesta, sleep duration at night, total sleep duration, and siesta ratio with hypertension or CVDs. Stratified analysis was performed in terms of age and sex. Variables with statistical difference between participants with hypertension and not as well as those with CVDs and not were regarded as confounding factors. To explore the associations between siesta, sleep duration at night, total sleep duration, and siesta ratio and hypertension, Model 1 was the unadjusted univariate logistic regression model, Model 2 was the multivariate logistical regression model adjusting for confounders including age, BMI, gender, marital status, education, and vigorous activity. To explore the associations between siesta, sleep duration at night, total sleep duration, and siesta ratio and CVDs, Model 1 was the unadjusted univariate logistic regression model, Model 2 was the multivariate logistical regression model adjusting for confounders including age, BMI, gender, registered permanent residence, education, and vigorous activity. The odds ratio (OR) and 95% confidence interval (CI) were applied for assessing the associations between siesta, sleep duration at night, total sleep duration, siesta ratio and hypertension or CVDs. All statistical analyses were conducted via SPSS software (IBM Corp.) and *p* < .05 referred to be statistically significant.

## 3 RESULTS

### 3.1 Comparisons of the baseline characteristic of patients with normal blood pressure and hypertension

**Figure 1:**
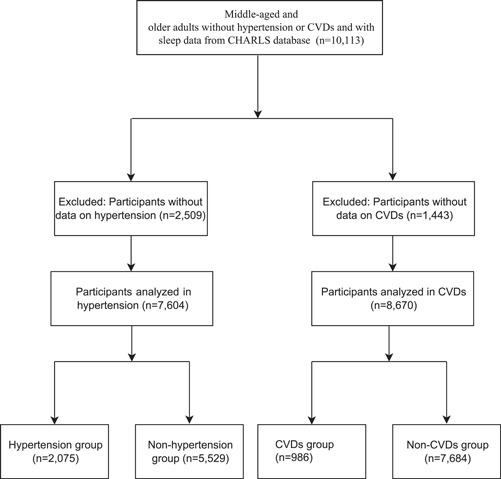
The screen process of the participants in this study. CVDs, cardiovascular diseases.

In this cohort study, 10 113 middle‐aged and older adults without hypertension or CVDs and with sleep data from CHARLS database were included. Among them 2509 participants were excluded as they lost the data on whether hypertension during follow‐up and the associations between siesta, sleep duration at night, total sleep duration, siesta ratio and hypertension were analyzed in 7604 participants. At the end of follow‐up, 2075 participants had hypertension. The detailed screen process was exhibited in Figure 1.

The baseline characteristics of patients with normal blood pressure and hypertension were exhibited in Table 1. The proration of people at different age group (*χ* ^2^ = 114.25, *p* < .001) and BMI group (*χ* ^2^ = 96.114, *p* < .001), participants with different gender (*χ* ^2^ = 9.817, *p* = .002), marital status (*χ* ^2^ = 25.511, *p* < .001), education levels (*χ* ^2^ = 42.441, *p* < .001), vigorous activity (*χ* ^2^ = 4.128, *p* = .042) were statistical different between subjects with normal blood pressure and hypertension. The percentages of people with different sleep duration at night (*χ* ^2^ = 7.812, *p* = .020), sleep duration (*χ* ^2^ = 8.854, *p* = .012) and people with different siesta ratio (*χ* ^2^ = 8.318, *p* = .040) in people with normal blood pressure and hypertension were statistically different.

**Table 1:** Comparisons of the baseline characteristic of patients with normal blood pressure and hypertension

Character	Hypertension (*n* = 2078)	Nonhypertension (*n* = 5541)	Statistical magnitude	*p*
Age, *n* (%)			*χ* ^2^ = 114.425	<.001
<60	1002 (48.29)	3421 (61.87)		
≥60	1073 (51.71)	2108 (38.13)		
BMI, *n* (%)			*χ* ^2^ = 96.114	<.001
<18.5	759 (36.58)	1583 (28.63)		
18.5−23.9	667 (32.14)	2455 (44.40)		
≥24	649 (31.28)	1491 (26.97)		
Gender, *n* (%)			*χ* ^2^ = 9.817	.002
Male	1030 (49.64)	2522 (45.61)		
Female	1045 (50.36)	3007 (54.39)		
Marital status, *n* (%)			*χ* ^2^ = 25.511	<.001
Married	1817 (87.57)	5046 (91.26)		
Others	38 (1.83)	90 (1.63)		
Widowed	220 (10.60)	393 (7.11)		
Registered permanent residence, *n* (%)			‐	.093
Agricultural residence	1695 (81.69)	4570 (82.66)		
Nonagricultural residence	345 (16.63)	903 (16.33)		
Unified Residence	34 (1.64)	54 (0.98)		
Do not have registered permanent residence	1 (0.05)	2 (0.04)		
Education, *n* (%)			*χ* ^2^ = 42.441	<.001
No formal education illiterate	541 (26.07)	1132 (20.47)		
Did not finish primary school but capable of reading or writing	333 (16.05)	882 (15.95)		
Elementary school	415 (20.00)	1076 (19.46)		
Middle school/High school/Vocational school	515 (24.82)	1493 (27.00)		
Two/Three‐Year College/Associate degree/Four‐Year College/Bachelor's degree/Post‐graduate, Master's degree	26 (1.25)	62 (1.12)		
Unknown/other	245 (11.81)	884 (15.99)		
Smoking, *n* (%)			*χ* ^2^ = 3.930	.140
Yes	104 (5.01)	329 (5.95)		
Quit smoke	61 (2.94)	134 (2.42)		
No	1298 (62.55)	3341 (60.43)		
Unknown	612 (29.49)	1725 (31.20)		
Drinking, *n* (%)			*χ* ^2^ = 1.139	.566
Yes (drink more than once a month)	118 (5.69)	347 (6.28)		
Yes (drink less than once a month)	50 (2.41)	143 (2.59)		
No	1295 (62.41)	3314 (59.95)		
Unknown	612 (29.49)	1725 (31.20)		
Vigorous activity, *n* (%)			*χ* ^2^ = 4.128	.042
Yes	269 (12.96)	818 (14.79)		
No	1806 (87.04)	4711 (85.21)		
Moderate activity, *n* (%)			*χ* ^2^ = 0.759	.384
Yes	415 (20.00)	1156 (20.91)		
No	1660 (80.00)	4373 (79.09)		
Walk activity, *n* (%)			*χ* ^2^ = 0.004	.950
Yes	599 (28.87)	1592 (28.79)		
No	1476 (71.13)	3937 (71.21)		
Dyslipidemia, *n* (%)			*χ* ^2^ = 0.835	.361
Yes	12 (0.58)	43 (0.78)		
No	2063 (99.42)	5486 (99.22)		
Diabetes mellitus dysglycemia, *n* (%)			*χ* ^2^ = 0.149	.699
Yes	12 (0.58)	28 (0.51)		
No	2063 (99.42)	5501 (99.49)		
CES‐D‐10, *n* (%)			*χ* ^2^ = 1.244	.265
Yes	210 (10.12)	513 (9.28)		
No	1865 (89.88)	5016 (90.72)		
Siesta, *n* (%)			*χ* ^2^ = 0.534	.465
Yes	1197 (57.69)	3138 (56.76)		
No	878 (42.31)	2391 (43.24)		
Siesta duration (h)			*χ* ^2^ = 6.541	.162
0	877 (42.27)	2508 (45.36)		
<0.5	7 (0.34)	14 (0.25)		
0.5 ≤ duration < 1	64 (3.08)	160 (2.89)		
1 ≤ duration < 1.5	9 (0.43)	29 (0.52)		
≥1.5	1118 (53.88)	2818 (50.97)		
Sleep duration at night (h)			*χ* ^2^ = 7.812	.020
<6	1155 (55.66)	2919 (52.79)		
6 ≤ duration < 8	772 (37.20)	2250 (40.69)		
≥8	148 (7.13)	360 (6.51)		
Sleep duration (h)			*χ* ^2^ = 8.854	.012
<6	824 (39.71)	2071 (37.46)		
6 ≤ duration < 8	773 (37.25)	2267 (41.00)		
≥8	478 (23.04)	1191 (21.54)		
Siesta ratio, *n* (%)			*χ* ^2^ = 8.318	.040
0	877 (42.27)	2508 (45.36)		
<0.2	877 (42.27)	2278 (41.20)		
0.2 ≤ duration < 0.4	302 (14.55)	704 (12.73)		
≥0.4	19 (0.92)	39 (0.71)		

Abbreviations: BMI, body mass index; CES‐D‐10: the 10‐item Center for Epidemiological Studies Depression Scale.

### 3.2 Associations of siesta, sleep duration at night, total sleep duration, siesta ratio with hypertension

In the univariate logistic model, people with sleep duration at night < 6 h (OR = 1.130, 95% CI: 1.006−1.270, *p* = .040), the total sleep duration < 6 h (OR = 1.176, 95% CI: 1.033−1.339, *p* = .014), the siesta ratio of 0 (OR = 0.850, 95% CI: 0.740−0.977, *p* = .023) and the siesta ratio ≥ 0.4 (OR = 1.742, 95% CI: 1.158−2.620, *p* = .008) might be associated with the risk of hypertension. After adjusting variables including marital status, gender, vigorous activity, age, education, and BMI (Model 2), total sleep duration < 6 h (OR = 1.168, 95% CI: 1.023−1.335, *p* = .022) and siesta ratios ≥ 0.4 (OR = 1.712, 95% CI: 1.129−2.594, *p* = .011) were risk factors for hypertension in middle‐aged and older adults (Table 2).

**Table 2:** Associations of siesta, sleep duration at night, total sleep duration, siesta ratio with hypertension

Variable	Model 1		Model 2	
	OR (95% CI)	*p*	OR (95% CI)	*p*
Siesta duration (h)		.044		.580
0	1.004 (0.841−1.200)	.961	1.017 (0.848−1.219)	.857
<0.5	1.037 (0.695−1.546)	.860	1.049 (0.700−1.572)	.818
0.5 ≤ duration < 1				
1 ≤ duration < 1.5	1.143 (0.945−1.382)	.169	1.095 (0.902−1.329)	.358
≥1.5	1.215 (0.999−1.477)	.051	1.123 (0.920−1.371)	.255
Sleep duration at night (h)		.101		.229
<6	1.130 (1.006−1.270)	.040	1.099 (0.975−1.238)	.123
6 ≤ duration < 8				
≥8	1.017 (0.895−1.157)	.793	0.995 (0.873−1.134)	.942
Sleep duration (h)		.049		.069
<6	1.176 (1.033−1.339)	.014	1.168 (1.023−1.335)	.022
6 ≤ duration < 8				
≥8	1.073 (0.954−1.206)	.238	1.049 (0.931‐1.181)	.435
Siesta ratio		.001		.024
0	0.850 (0.740−0.977)	.023	0.924 (0.802−1.065)	.277
<0.2	0.920 (0.797−1.062)	.253	0.956 (0.826−1.106)	.546
0.2 ≤ duration < 0.4				
≥0.4	1.742 (1.158−2.620)	.008	1.712 (1.129−2.594)	.011

*Note*: Model 1: unadjusted model; Model 2: multivariate regression model adjusting for age, BMI, gender, marital status, education, and vigorous activity.

In males aged ≥ 60 years, the multivariate logistic regression depicted that the siesta ratio ≥ 0.4 was linked with higher risk of hypertension (OR = 3.009, 95% CI: 1.313−6.899, *p* = .009). In males aged < 60 years, the total sleep duration < 6 h might be associated with the risk of hypertension (OR = 1.337, 95% CI: 1.011−1.768, *p* = .042) and multivariate analysis delineated that the risk of hypertension was increased by 0.363 time in people with the total sleep duration < 6 h (OR = 1.363, 95% CI: 1.026−1.809, *p* = .032). As for females aged ≥ 60 years and <60 years, no significant association was observed between siesta, sleep duration at night, total sleep duration, siesta ratio and hypertension (all *p* > .05) (Supporting Information: Table 1).

### 3.3 Comparisons of the baseline characteristic of patients with or without CVDs

In total, 10 113 middle‐aged and older adults without hypertension or CVDs and with sleep data from CHARLS database were included. Among them, 1443 subjects lost the data on CVDs during the follow‐up and the associations between siesta, sleep duration at night, total sleep duration, siesta ratio and CVDs were analyzed in 8670 participants. At the end of follow‐up, 986 people had CVDs. The detailed screen process was exhibited in Figure 1.

As displayed in Table 3, the proration of patients at different age group (*χ* ^2^ = 41.093, *p* < .001), BMI group (*χ* ^2^ = 16.927 *p* < .001), patients with different genders (*χ* ^2^ = 23.486, *p* = .002), registered permanent residence (*χ* ^2^ = 15.595, *p* = .001), education (*χ* ^2^ = 15.399, *p* = .009), vigorous activity (*χ* ^2^ = 6.396, *p* = .011), siesta (*χ* ^2^ = 7.086, *p* = .008), sleep duration at night (*χ* ^2^ = 24.879, *p* < .001), and total sleep duration (*χ* ^2^ = 21.674, *p* < .001) were statistically different between patients with CVDs and patients without CVDs.

**Table 3:** Comparisons of the baseline characteristic of patients with or without CVDs

Character	CVDs (*n* = 986)	Non‐CVDs (*n* = 7684)	Statistical magnitude	*p*
Age, *n* (%)			*χ* ^2^ = 41.093	<.001
<60	508 (51.52)	4772 (62.10)		
≥60	478 (48.48)	2912 (37.90)		
BMI, *n* (%)			*χ* ^2^ = 16.927	<.001
<18.5	312 (31.64)	2492 (32.43)		
18.5−23.9	348 (35.29)	3106 (40.42)		
≥24	326 (33.06)	2086 (27.15)		
Gender, n (%)			*χ* ^2^ = 23.486	<.001
Male	389 (39.45)	3660 (47.63)		
Female	597 (60.55)	4024 (52.37)		
Marriage, *n* (%)			*χ* ^2^ = 3.320	.190
Married	884 (89.66)	7007 (91.19)		
Others	17 (1.72)	137 (1.78)		
Widowed	85 (8.62)	540 (7.03)		
Registered permanent residence, *n* (%)			*χ* ^2^ = 15.595	.001
Agricultural residence	764 (77.48)	6260 (81.47)		
Nonagricultural residence	216 (21.91)	1328 (17.28)		
Unified residence	6 (0.61)	93 (1.21)		
Do not have registered permanent residence	0 (0.00)	3 (0.04)		
Education, *n* (%)			*χ* ^2^ = 15.399	.009
No formal education illiterate	220 (22.31)	1579 (20.55)		
Did not finish primary school but capable of reading or writing	159 (16.13)	1173 (15.27)		
Elementary school	202 (20.49)	1446 (18.82)		
Middle school/High school/Vocational school	270 (27.38)	2078 (27.04)		
Two/Three‐Year College/Associate degree/Four‐Year College/Bachelor's degree/Post‐graduate, Master's degree	15 (1.52)	105 (1.37)		
Unknown/other	120 (12.17)	1303 (16.96)		
Smoking, *n* (%)			*χ* ^2^ = 4.826	.090
Yes	46 (4.67)	492 (6.40)		
Quit smoke	30 (3.04)	207 (2.69)		
No	579 (58.72)	4672 (60.80)		
Unknown	331 (33.57)	2313 (30.10)		
Drinking, *n* (%)			*χ* ^2^ = 0.123	.941
Yes (drink more than once a month)	64 (6.49)	492 (6.40)		
Yes (drink less than once a month)	24 (2.43)	201 (2.62)		
No	567 (57.51)	4678 (60.90)		
Unknown	331 (33.57)	2313 (30.10)		
Vigorous activity, *n* (%)			*χ* ^2^ = 6.396	.011
Yes	116 (11.76)	1135 (14.77)		
No	870 (88.24)	6549 (85.23)		
Moderate activity, *n* (%)			*χ* ^2^ = 1.502	.220
Yes	193 (19.57)	1634 (21.26)		
No	793 (80.43)	6050 (78.74)		
Walk activity, *n* (%)			*χ* ^2^ = 0.043	.835
Yes	283 (28.70)	2230 (29.02)		
No	703 (71.30)	5454 (70.98)		
Dyslipidemia, *n* (%)			*χ* ^2^ = 0.395	.530
Yes	10 (1.01)	63 (0.82)		
No	976 (98.99)	7621 (99.18)		
Diabetes mellitus dysglycemia, *n* (%)			*χ* ^2^ = 0.012	.914
Yes	5 (0.51)	41 (0.53)		
No	981 (99.49)	7643 (99.47)		
CES‐D‐10, *n* (%)			*χ* ^2^ = 0.045	.831
Yes	92 (9.33)	701 (9.12)		
No	894 (90.67)	6983 (90.88)		
Siesta, *n* (%)			*χ* ^2^ = 7.086	.008
Yes	602 (61.05)	4349 (56.60)		
No	384 (38.95)	3335 (43.40)		
Siesta duration (h)			*χ* ^2^ = 4.254	.373
0	432 (43.81)	3465 (45.09)		
<0.5	5 (0.51)	19 (0.25)		
0.5 ≤ duration < 1	27 (2.74)	204 (2.65)		
1 ≤ duration < 1.5	8 (0.81)	34 (0.44)		
≥1.5	514 (52.13)	3962 (51.56)		
Sleep duration at night (h)			*χ* ^2^ = 24.879	<.001
<6	604 (61.26)	4068 (52.94)		
6≤duration<8	323 (32.76)	3112 (40.50)		
≥8	59 (5.98)	504 (6.56)		
Sleep duration (h)			χ^2^ = 21.674	<.001
<6	444 (45.03)	2873 (37.39)		
6 ≤ duration < 8	357 (36.21)	3139 (40.85)		
≥8	185 (18.76)	1672 (21.76)		
Siesta ratio, *n* (%)			*χ* ^2^ = 1.413	.702
0	432 (43.81)	3465 (45.09)		
<0.2	413 (41.89)	3184 (41.44)		
0.2 ≤ duration < 0.4	131 (13.29)	978 (12.73)		
≥0.4	10 (1.01)	57 (0.74)		

Abbreviations: BMI, body mass index; CES‐D‐10: the 10‐item Center for Epidemiological Studies Depression Scale.

### 3.4 Associations between siesta, sleep duration at night, total sleep duration, the ratio of siesta in total sleep duration, and CVDs

In Model 1, people with siesta duration <0.5 h (OR = 2.104, 95% CI: 1.361−3.253, *p* = .001), people with sleep duration at night < 6 h (OR = 1.458, 95% CI: 1.256−1.692, *p* < .001) and total sleep duration < 6 h (OR = 1.339, 95% CI: 1.138−1.576, *p* < .001) might be associated with the risk of CVDs. When age, BMI, gender, registered permanent residence, education, and vigorous activity were adjusted, the risk of CVDs was elevated by 1.053 times in people with siesta duration <0.5 h (OR = 2.053, 95% CI: 1.323−3.185, *p* = .001). Increased risk of CVDs was also observed in people sleep duration at night < 6 h (OR = 1.374, 95% CI: 1.180−1.599, *p* < .001) and total sleep duration < 6 h (OR = 1.260, 95% CI: 1.068−1.488, *p* = .006) after adjusting marital status, gender, vigorous activity, age, education, and BMI (Table 4).

**Table 4:** Associations between siesta, sleep duration at night, total sleep duration, the ratio of siesta in total sleep duration, and CVDs

Variable	Model 1		Model 2	
	OR (95% CI)	*p*	OR (95% CI)	*p*
Siesta duration (h)		.016		.023
0	1.090 (0.861−1.379)	.474	1.106 (0.872−1.403)	.408
<0.5	2.104 (1.361−3.253)	.001	2.053 (1.323−3.185)	.001
0.5 ≤ duration < 1				
1≤duration<1.5	1.127 (0.875−1.451)	.356	1.156 (0.896−1.493)	.265
≥1.5	1.172 (0.903−1.521)	.233	1.208 (0.928−1.573)	.161
Sleep duration at night (h)		<.001		<.001
<6	1.458 (1.256−1.692)	<.001	1.374 (1.180−1.599)	<.001
6 ≤ duration < 8				
≥8	0.921 (0.770−1.102)	.371	0.908 (0.758−1.088)	.296
Sleep duration (h)		<.001		<.001
<6	1.339 (1.138−1.576)	<.001	1.260 (1.068−1.488)	.006
6 ≤ duration < 8				
≥8	0.886 (0.757−1.039)	.136	0.889 (0.758−1.043)	.149
Siesta ratio		.707		.718
0	0.900 (0.749−1.080)	.256	0.896 (0.745−1.079)	.247
<0.2	0.921 (0.763−1.112)	.394	0.927 (0.766−1.122)	.437
0.2 ≤ duration < 0.4				
≥0.4	1.009 (0.565−1.802)	.975	0.953 (0.532−1.709)	.873

*Note*: Model 1: unadjusted model; Model 2: multivariate regression model adjusting for age, BMI, gender, registered permanent residence, education and vigorous activity.

With regard to males ≥ 60 or <60 years old, no statistical association was observed between siesta duration, sleep duration at night, total sleep duration, siesta ratio, and CVDs (all *p* > .05). As for females ≥ 60 years, the sleep duration at night < 6 h was associated with increased risk of CVDs (OR = 1.617, 95% CI: 1.187−2.204, *p* = .002) after adjusting confounders. The risk of CVDs was increased by 0.388 times in females ≥ 60 years with the total sleep duration < 6 h (OR = 1.388, 95% CI: 1.004−1.918, *p* = .047) after adjusting marital status, gender, vigorous activity, age, education and BMI. In females < 60 years, after adjusting those confounders, those with siesta duration < 0.5 h were associated with 2.946‐fold risk of CVDs (OR = 2.946, 95% CI: 1.529−5.673, *p* = .001). Participants with sleep duration at night < 6 h was associated with a higher risk of CVDs with an adjusted OR of 1.376 (OR = 1.376, 95% CI: 1.065−1.778, *p* = .015). People with sleep duration at night ≥ 8 h (OR = 0.714, 95% CI: 0.518−0.985, *p* = .040) and total sleep duration ≥ 8 h (OR = 0.594, 95% CI: 0.445−0.793, *p* < .001) were correlated with a lower risk of CVDs (Supporting Information: Table 2).

## 4 DISCUSSION

In this study, the associations between siesta, sleep duration at night, total sleep duration, siesta ratio and hypertension were analyzed in 7604 participants while the associations between siesta, sleep duration at night, total sleep duration, siesta ratio, and CVDs were analyzed in 8670 participants. The results showed that in the total population, sleep duration < 6 h and siesta ratio ≥ 0.4 were associated with higher risk of hypertension. Subgroup analysis depicted that in males ≥ 60 years, siesta ratio ≥ 0.4 was linked with higher risk of hypertension while in males < 60 years, total sleep duration < 6 h was associated with increased risk of hypertension. In the total population, the siesta duration < 0.5 h, sleep duration at night < 6 h and total sleep duration < 6 h were correlated with elevated risk of CVDs. Sleep duration at night < 6 h and total sleep duration < 6 h were associated with increased risk of CVDs in females ≥ 60 years. Siesta duration < 0.5 h, and sleep duration at night < 6 h were associated with increased risk of CVDs while sleep duration at night ≥ 8 h and total sleep duration ≥ 8 h were correlated with decreased risk of CVDs in females < 60 years.

In previous studies, multiply studies have demonstrated that total sleep duration was associated with the risk of hypertension in people. Tang et al. explored the association between sleep duration and the incidence of hypertension in the elderly aged ≥ 65 years old and found the risk of hypertension was associated with 1.37 times in those with sleep duration < 6 h compared with those with sleep duration of 6−7 h.^18^ Another meta‐analysis involving 11 publications with 85 838 subjects also indicated that short sleep duration was a risk factor for the incidence of hypertension.^19^ These findings supported the results in our study, which depicted that the sleep duration < 6 h was correlated with increased risk of hypertension in old adults compared with the sleep duration 6–8 h. For people aged ≥ 45 years, sufficient sleep was recommended. Another finding in our study was that the ratio of siesta duration in total sleep duration ≥ 0.4 might be a risk factor for hypertension. A cohort study in China identified that the levels of SBP and DBP were increased with longer duration of habitual midday napping.^20^ A longer siesta may increase the body's stress response and lead to short sleep, poor sleep quality, which may contribute to high blood pressure.^20^, ^21^ A longer daytime naps may lead to the elevation of evening cortisol levels and then increase the blood pressure levels.^22^ A high ratio of siesta suggested a long duration of siesta and a shorter duration of the sleep at night.^23^ The mechanisms of these might because a longer duration of siesta may result in insomnia or low quality of sleep at night. Increasing studies indicated that a better night sleep is essential for the health of people.^24^ For people aged ≥ 45 years, long duration of afternoon nap should be avoided, they were recommended to have longer night sleep instead of afternoon nap. Subgroup analysis exhibited that siesta ratio ≥ 0.4 was linked with higher risk of hypertension in males ≥ 60 years while total sleep duration < 6 was associated with increased risk of hypertension in males < 60 years. Usually, the prevalence of hypertension was higher in men than in women.^25^ Differences also exist between males and females concerning lifestyles including drinking or smoking habits.^26^ For males ≥ 60 years, higher risk of hypertension was observed in those with high afternoon nap ratio, and to decrease the risk of hypertension, the duration of afternoon nap should be controlled.

The association between siesta and the risk of CVDs has been explored by many research. Mohammad explored that the practice of regular siesta could reduce the risk of ischemic stroke.^13^ Bursztyn et al. found that people with siesta duration < 1 h was associated with higher risk of mortality with an OR of 4.67.^27^ Krittanawong et al. indicated that short (<7 h) and long sleep durations (>9 h) increased the risk of overall mortality of CVDs, particularly in Asian populations and elderly individuals.^28^ In the current study, higher risk of CVDs was observed in people with the siesta duration < 0.5 h. This may be due to that the fragmented sleep is associated with decreased physical activity, insulin resistance, obesity, and increased inflammation in people.^29^, ^30^ Siesta was also reported to be associated with higher level of C‐reactive protein.^31^ For people who preferred siesta, more than half an hour was recommended. Additionally, sleep duration at night < 6 h and total sleep duration < 6 h were correlated with an elevated risk of CVDs. There was evidence showing that people who have insufficient sleep was associated with the risk of CVDs.^32^ St‐Onge et al. published an article which revealed that sleep duration, mostly short sleep, are related to adverse cardiometabolic risk including CVDs.^33^ These gave support the findings of our study. Evidence suggests that sleep deprivation might result in inflammation and sufficient sleep might help the immune cytokines return to the baseline.^34^ Sleep can profoundly modify the cardiovascular regulation, and a bidirectional link exists in the interconnection between cardiovascular system and sleep.^35^ Another intriguing finding in the present study was that the sleep duration at night ≥ 8 h and total sleep duration ≥ 8 h were correlated with decreased risk of CVDs in females < 60 years. Previously, most studies proposed that people with long duration of sleep may elevate the incidence of CVDs.^36^ Different results obtained in our study might because the age in females was <60 years, which were mostly postmenopausal women, and the sleep quality was poor in those people.^37^ For females aged 45−60 years, enough sleep might be beneficial for the stable hormone levels and health status. There was evidence revealed that obstructive sleep apnea was associated with an increased risk of CVDs,^38^, ^39^ and proper interventions should be provided to those with obstructive sleep apnea for the potential of prevention for CVDs.

This study evaluated the associations between siesta, sleep duration at night, total sleep duration, the percentage of siesta in total sleep duration and hypertension or CVDs with large scale of sample size. We initially explored the association of siesta duration/total sleep duration ratio and hypertension or CVDs. The findings of this study implied that 6−8 h sleep a day especially nighttime sleep were required for all the population, but too long siesta duration was associated with higher risk for hypertension especially males. Meanwhile, siesta duration < 0.5 h should be avoided, and for women aged < 60 years, sleep duration ≥ 8 h might be good for decreasing the risk of CVDs.

In the current study, the first limitation was that we could only obtain the correlations between siesta, sleep duration at night, total sleep duration, siesta ratio and hypertension or CVDs. Second, some important variables including sleep duration were self‐reported, which might cause bias. Thirdly, the factors influencing the risk of hypertension or CVDs were various, and some variables were not included in the CHARLS database. Further studies are warranted on this topic to validate the findings in this study.

## 5 CONCLUSIONS

Herein, the associations between siesta, sleep duration at night, total sleep duration, the percentage of siesta in total sleep duration and hypertension or CVDs were analyzed. The results showed that short sleep duration and high siesta ratio were associated with higher risk of hypertension. Only associations were obtained without any evidence of causality. In males ≥ 60 years, high siesta ratio was linked with higher risk of hypertension while in males < 60 years, short sleep duration was associated with increased risk of hypertension. Short siesta duration, sleep duration at night and total sleep duration were correlated with an elevated risk of CVDs. Short sleep duration at night and total sleep duration were associated with increased risk of CVDs in females ≥ 60 years. Short siesta duration and sleep duration at night were associated with increased risk of CVDs in females < 60 years. The findings of the current study might provide a reference for the siesta and sleep duration in people aged ≥ 45 years and more rational sleep plan should be made. The future scope of our study will be exploring the associations between sleep quality with the risk of arterial hypertension and CVDs.

## CONFLICT OF INTEREST

The authors declare no conflict of interest.

## Supporting information

Supporting information.

## Data Availability

The datasets used and/or analyzed during the current study are available from the corresponding author on reasonable request.

## References

[1] Murray C , Aravkin AY , Zheng P , et al. Global burden of 87 risk factors in 204 countries and territories, 1990‐2019: a systematic analysis for the global burden of disease study 2019. The Lancet. 2020;396(10258):1223‐1249. 10.1016/S0140-6736(20)30752-2 PMC7566194 33069327

[2] Mills KT , Bundy JD , Kelly TN , et al. Global disparities of hypertension prevalence and control: a systematic analysis of Population‐Based studies from 90 countries. Circulation. 2016;134(6):441‐450. 27502908 10.1161/CIRCULATIONAHA.115.018912 PMC4979614

[3] Mancia G . Introduction to a compendium on hypertension. Circ Res. 2015;116(6):923‐924. 25767280 10.1161/CIRCRESAHA.115.305755

[4] Fan F , Yuan Z , Qin X , et al. Optimal systolic blood pressure levels for primary prevention of stroke in general hypertensive adults: findings from the CSPPT (China stroke primary prevention trial). Hypertension. 2017;69(4):697‐704. 28242714 10.1161/HYPERTENSIONAHA.116.08499

[5] Bahbah EI , Noehammer C , Pulverer W , Jung M , Weinhaeusel A . Salivary biomarkers in cardiovascular disease: an insight into the current evidence. FEBS J. 2021;288(22):6392‐6405. 33370493 10.1111/febs.15689

[6] Roth GA , Mensah GA , Johnson CO , et al. Global burden of cardiovascular diseases and risk factors, 1990–2019. J Am Coll Cardiol. 2020;76(25):2982‐3021. 33309175 10.1016/j.jacc.2020.11.010 PMC7755038

[7] Lenfant C , Chobanian AV , Jones DW , Roccella EJ . Seventh report of the joint national committee on the prevention, detection, evaluation, and treatment of high blood pressure (JNC 7): resetting the hypertension sails. Hypertension. 2003;41(6):1178‐1179. 12756222 10.1161/01.HYP.0000075790.33892.AE

[8] Bathgate CJ , Fernandez‐Mendoza J . Insomnia, short sleep duration, and high blood pressure: recent evidence and future directions for the prevention and management of hypertension. Curr Hypertens Rep. 2018;20(6):52. 29779139 10.1007/s11906-018-0850-6

[9] Han B , Chen WZ , Li YC , Chen J , Zeng ZQ . Sleep and hypertension. Sleep Breath. 2020;24(1):351‐356. 31402441 10.1007/s11325-019-01907-2 PMC7127991

[10] Bertisch SM , Pollock BD , Mittleman MA , et al. Insomnia with objective short sleep duration and risk of incident cardiovascular disease and all‐cause mortality: sleep heart health study. Sleep. 2018;41(6):zsy047. 29522193 10.1093/sleep/zsy047 PMC5995202

[11] Zhong G , Wang Y , Tao T , Ying J , Zhao Y . Daytime napping and mortality from all causes, cardiovascular disease, and cancer: a meta‐analysis of prospective cohort studies. Sleep Med. 2015;16(7):811‐819. 26051864 10.1016/j.sleep.2015.01.025

[12] Zhou Y , Wang Z , Lu J , et al. Effects of nighttime sleep duration and sex on the association between siesta and hypertension. Sleep Med. 2021;82:200‐209. 33957416 10.1016/j.sleep.2021.04.005

[13] Mohammad Y . Siesta and risk for ischemic stroke: results from a Case‐Control study. Medicina. 2020;56:222. 32392748 10.3390/medicina56050222 PMC7279277

[14] Cao YM , Li D , Li KB , et al. [Epidemiological study on the relationship between the siesta and blood pressure]. Zhonghua Yi Xue Za Zhi. 2016;96(21):1699‐1701. 27290714 10.3760/cma.j.issn.0376-2491.2016.21.018

[15] Al‐Abri MA , Al Lawati I , Zadjali F , Ganguly S . Sleep patterns and quality in Omani adults. Nat Sci Sleep. 2020;12:231‐237. 32341667 10.2147/NSS.S233912 PMC7166064

[16] Tsiptsios D , Matziridis A , Ouranidis A , et al. Age and gender effects on the association of sleep insufficiency with hypertension among adults in Greece. Future Cardiol. 2021;17(8):1381‐1393. 33646018 10.2217/fca-2020-0198

[17] Zhao Y , Hu Y , Smith JP , Strauss J , Yang G . Cohort profile: the China health and retirement longitudinal study (CHARLS). Int J Epidemiol. 2014;43(1):61‐68. 23243115 10.1093/ije/dys203 PMC3937970

[18] Tang ML , Wei F , Zhang HF , et al. [Association between sleep and prevalence of hypertension in elderly population]. Zhonghua Liu Xing Bing Xue Za Zhi. 2021;42(7):1188‐1193. 34814529 10.3760/cma.j.cn112338-20200512-00713

[19] Wang L , Hu Y , Wang X , Yang S , Chen W , Zeng Z . The association between sleep duration and hypertension: a meta and study sequential analysis. J Hum Hypertens. 2021;35(7):621‐626. 32587332 10.1038/s41371-020-0372-y

[20] Cao Z , Shen L , Wu J , et al. The effects of midday nap duration on the risk of hypertension in a middle‐aged and older Chinese population: a preliminary evidence from the Tongji‐Dongfeng cohort study, China. J Hypertens. 2014;32(10):1993‐1998; discussion 1998. 25023156 10.1097/HJH.0000000000000291

[21] Castro‐Diehl C , Diez Roux AV , Redline S , et al. Sleep duration and quality in relation to autonomic nervous system measures: the Multi‐Ethnic study of atherosclerosis (MESA). Sleep. 2016;39(11):1927‐1940. 27568797 10.5665/sleep.6218 PMC5070747

[22] Woods DL , Kim H , Yefimova M . To nap or not to nap: excessive daytime napping is associated with elevated evening cortisol in nursing home residents with dementia. Biol Res Nurs. 2013;15(2):185‐190. 21998447 10.1177/1099800411420861

[23] Ohayon M , Wickwire EM , Hirshkowitz M , et al. National sleep foundation's sleep quality recommendations: first report. Sleep Health. 2017;3(1):6‐19. 28346153 10.1016/j.sleh.2016.11.006

[24] Manolis TA , Manolis AA , Apostolopoulos EJ , Melita H , Manolis AS . Cardiovascular complications of sleep disorders: a better night's sleep for a healthier Heart/From bench to bedside. Curr Vasc Pharmacol. 2020;19(2):210‐232. 10.2174/1570161118666200325102411 32209044

[25] Bao M , Wang L . The longitudinal trend of hypertension prevalence in Chinese adults from 1959 to 2018: a systematic review and meta‐analysis. Ann Palliat Med. 2020;9(5):2485‐2497. 32819115 10.21037/apm-19-377

[26] Basit A , Tanveer S , Fawwad A , Naeem N . Prevalence and contributing risk factors for hypertension in urban and rural areas of Pakistan; a study from second National Diabetes Survey of Pakistan (NDSP) 2016‐2017. Clin Exp Hypertens. 2020;42(3):218‐224. 31151358 10.1080/10641963.2019.1619753

[27] Bursztyn M , Ginsberg G , Stessman J . The siesta and mortality in the elderly: effect of rest without sleep and daytime sleep duration. Sleep. 2002;25(2):187‐191. 11902427 10.1093/sleep/25.2.187

[28] Krittanawong C , Tunhasiriwet A , Wang Z , et al. Association between short and long sleep durations and cardiovascular outcomes: a systematic review and meta‐analysis. Eur Heart J Acute Cardiovasc Care. 2019;8(8):762‐770. 29206050 10.1177/2048872617741733

[29] Cox NS , Pepin V , Holland AE . Greater sleep fragmentation is associated with less physical activity in adults with cystic fibrosis. J Cardiopulm Rehabil Prev. 2019;39(1):E11‐E14. 30252784 10.1097/HCR.0000000000000363

[30] Yan B , Zhao B , Fan Y , et al. The association between sleep efficiency and diabetes mellitus in community‐dwelling individuals with or without sleep‐disordered breathing. J Diabetes. 2020;12(3):215‐223. 31503406 10.1111/1753-0407.12987

[31] Leng Y , Ahmadi‐Abhari S , Wainwright NWJ , et al. Daytime napping, sleep duration and serum C reactive protein: a population‐based cohort study. BMJ Open. 2014;4(11):e006071. 10.1136/bmjopen-2014-006071 PMC4244397 25387759

[32] Khan MS , Aouad R . The effects of insomnia and sleep loss on cardiovascular disease. Sleep Med Clin. 2017;12(2):167‐177. 28477772 10.1016/j.jsmc.2017.01.005

[33] St‐Onge MP , Grandner MA , Brown D , et al. Sleep duration and quality: impact on lifestyle behaviors and cardiometabolic health: a scientific statement from the American heart association. Circulation. 2016;134(18):e367‐e386. 27647451 10.1161/CIR.0000000000000444 PMC5567876

[34] Mantua J , Spencer RMC . Exploring the nap paradox: are mid‐day sleep bouts a friend or foe? Sleep Med. 2017;37:88‐97. 28899546 10.1016/j.sleep.2017.01.019 PMC5598771

[35] Tobaldini E , Costantino G , Solbiati M , et al. Sleep, sleep deprivation, autonomic nervous system and cardiovascular diseases. Neurosci Biobehav Rev. 2017;74(Pt B):321‐329. 27397854 10.1016/j.neubiorev.2016.07.004

[36] Matsubayashi H , Nagai M , Dote K , et al. Long sleep duration and cardiovascular disease: associations with arterial stiffness and blood pressure variability. J Clin Hypertens. 2021;23(3):496‐503. 10.1111/jch.14163 PMC8029549 33377597

[37] Kalmbach DA , Cheng P , Arnedt JT , et al. Treating insomnia improves depression, maladaptive thinking, and hyperarousal in postmenopausal women: comparing cognitive‐behavioral therapy for insomnia (CBTI), sleep restriction therapy, and sleep hygiene education. Sleep Med. 2019;55:124‐134. 30785053 10.1016/j.sleep.2018.11.019 PMC6503531

[38] McEvoy RD , Antic NA , Heeley E , et al. CPAP for prevention of cardiovascular events in obstructive sleep apnea. N Engl J Med. 2016;375(10):919‐931. 27571048 10.1056/NEJMoa1606599

[39] Yu J , Zhou Z , McEvoy RD , et al. Association of positive airway pressure with cardiovascular events and death in adults with sleep apnea: a systematic review and meta‐analysis. JAMA. 2017;318(2):156‐166. 28697252 10.1001/jama.2017.7967 PMC5541330

