# Supplementary material for: Associations of siesta and total sleep duration with hypertension or cardiovascular diseases in middle‐aged and older adults
Source: Clin Cardiol. 2022 Dec 12;46(2):159–70. doi: 10.1002/clc.23954 (PMC9933105; doi:10.1002/clc.23954)
Supplement: Supplementary file 1 — Supporting information. [file CLC-46-159-s001.docx]

Supplementary Table 1 Associations of siesta, sleep duration at night, total sleep duration, siesta ratio with hypertension in different subgroup

| Variable | Model 1 | | Model 2 | |
| --- | --- | --- | --- | --- |
|  | OR (95%CI) | *P* | OR (95%CI) | *P* |
| Male ≥60 years |  |  |  |  |
| Siesta duration (h) |  | 0.217 |  | 0.412 |
| 0 | 0.981 (0.649-1.483) | 0.928 | 1.096 (0.716-1.676) | 0.674 |
| <0.5 | 1.229 (0.455-3.317) | 0.684 | 1.318 (0.480-3.621) | 0.592 |
| 0.5≤duration<1 |  |  |  |  |
| 1≤duration<1.5 | 1.212 (0.793-1.853) | 0.374 | 1.235 (0.799-1.908) | 0.342 |
| ≥1.5 | 1.307 (0.857-1.994) | 0.213 | 1.377 (0.893-2.123) | 0.148 |
| Sleep duration at night (h) |  | 0.630 |  | 0.651 |
| <6 | 0.928 (0.728-1.182) | 0.543 | 0.947 (0.741-1.212) | 0.667 |
| 6≤duration<8 |  |  |  |  |
| ≥8 | 1.059 (0.823-1.362) | 0.656 | 1.080 (0.836-1.395) | 0.557 |
| Sleep duration (h) |  | 0.471 |  | 0.476 |
| <6 | 0.953 (0.719-1.264) | 0.740 | 0.990 (0.743-1.317) | 0.943 |
| 6≤duration<8 |  |  |  |  |
| ≥8 | 1.114 (0.886-1.401) | 0.354 | 1.136 (0.900-1.434) | 0.284 |
| Siesta ratio |  | 0.009 |  | 0.026 |
| 0 | 0.818 (0.624-1.072) | 0.146 | 0.864 (0.657-1.137) | 0.297 |
| <0.2 | 0.986 (0.755-1.287) | 0.915 | 0.951 (0.725-1.247) | 0.716 |
| 0.2≤duration<0.4 |  |  |  |  |
| ≥0.4 | 3.044 (1.344-6.893) | 0.008 | 3.009 (1.313-6.899) | 0.009 |
| Males < 60 years |  |  |  |  |
| Siesta duration (h) |  | 0.753 |  | 0.712 |
| 0 | 1.067 (0.748-1.522) | 0.722 | 1.111 (0.775-1.594) | 0.567 |
| <0.5 | 0.798 (0.308-2.068) | 0.642 | 0.864 (0.330-2.260) | 0.766 |
| 0.5≤duration<1 |  |  |  |  |
| 1≤duration<1.5 | 1.121 (0.773-1.627) | 0.547 | 1.112 (0.763-1.621) | 0.582 |
| ≥1.5 | 0.931 (0.628-1.380) | 0.722 | 0.924 (0.620-1.378) | 0.700 |
| Sleep duration at night (h) |  | 0.136 |  | 0.118 |
| <6 | 1.226 (0.962-1.564) | 0.100 | 1.222 (0.955-1.564) | 0.111 |
| 6≤duration<8 |  |  |  |  |
| ≥8 | 0.936 (0.718-1.219) | 0.622 | 0.911 (0.697-1.190) | 0.494 |
| Sleep duration (h) |  | 0.081 |  | 0.052 |
| <6 | 1.337 (1.011-1.768) | 0.042 | 1.363 (1.026-1.809) | 0.032 |
| 6≤duration<8 |  |  |  |  |
| ≥8 | 0.997 (0.790-1.259) | 0.982 | 0.979 (0.773-1.240) | 0.860 |
| Siesta ratio |  | 0.677 |  | 0.727 |
| 0 | 1.118 (0.832-1.502) | 0.460 | 1.151 (0.854-1.551) | 0.356 |
| <0.2 | 1.091 (0.812-1.466) | 0.564 | 1.070 (0.793-1.443) | 0.658 |
| 0.2≤duration<0.4 |  |  |  |  |
| ≥0.4 | 1.726 (0.667-4.468) | 0.261 | 1.467 (0.556-3.871) | 0.438 |
| Females ≥60 years |  |  |  |  |
| Siesta duration (h) |  | 0.360 |  | 0.354 |
| 0 | 0.837 (0.588-1.191) | 0.323 | 0.822 (0.573-1.179) | 0.286 |
| <0.5 | 1.277 (0.597-2.731) | 0.528 | 1.325 (0.615-2.854) | 0.472 |
| 0.5≤duration<1 |  |  |  |  |
| 1≤duration<1.5 | 0.859 (0.583-1.267) | 0.444 | 0.844 (0.569-1.252) | 0.400 |
| ≥1.5 | 1.078 (0.718-1.618) | 0.718 | 1.047 (0.694-1.581) | 0.826 |
| Sleep duration at night (h) |  | 0.350 |  | 0.441 |
| <6 | 1.182 (0.927-1.505) | 0.177 | 1.165 (0.910-1.492) | 0.224 |
| 6≤duration<8 |  |  |  |  |
| ≥8 | 1.026 (0.770-1.365) | 0.862 | 1.034 (0.774-1.382) | 0.821 |
| Sleep duration (h) |  | 0.226 |  | 0.222 |
| <6 | 1.255 (0.967-1.629) | 0.088 | 1.258 (0.964-1.640) | 0.091 |
| 6≤duration<8 |  |  |  |  |
| ≥8 | 1.163 (0.893-1.514) | 0.262 | 1.181 (0.904-1.543) | 0.222 |
| Siesta ratio |  | 0.259 |  | 0.322 |
| 0 | 0.806 (0.606-1.073) | 0.140 | 0.812 (0.607-1.086) | 0.161 |
| <0.2 | 0.874 (0.644-1.186) | 0.387 | 0.888 (0.652-1.210) | 0.452 |
| 0.2≤duration<0.4 |  |  |  |  |
| ≥0.4 | 1.447 (0.663-3.159) | 0.353 | 1.400 (0.636-3.078) | 0.403 |
| Females <60 years |  |  |  |  |
| Siesta duration (h) |  | 0.614 |  | 0.516 |
| 0 | 1.125 (0.806-1.569) | 0.489 | 1.152 (0.823-1.612) | 0.410 |
| <0.5 | 1.008 (0.502-2.002) | 0.983 | 0.983 (0.487-1.983) | 0.962 |
| 0.5≤duration<1 |  |  |  |  |
| 1≤duration<1.5 | 1.239 (0.858-1.790) | 0.252 | 1.312 (0.905-1.902) | 0.151 |
| ≥1.5 | 1.308 (0.889-1.925) | 0.173 | 1.311 (0.887-1.938) | 0.174 |
| Sleep duration at night (h) |  | 0.867 |  | 0.785 |
| <6 | 1.042 (0.834-1.303) | 0.717 | 1.036 (0.826-1.299) | 0.761 |
| 6≤duration<8 |  |  |  |  |
| ≥8 | 0.973 (0.762-1.242) | 0.826 | 0.944 (0.737-1.209) | 0.646 |
| Sleep duration (h) |  | 0.724 |  | 0.633 |
| <6 | 1.069 (0.839-1.362) | 0.588 | 1.071 (0.838-1.368) | 0.584 |
| 6≤duration<8 |  |  |  |  |
| ≥8 | 0.966 (0.771-1.210) | 0.763 | 0.946 (0.753-1.188) | 0.633 |
| Siesta ratio |  | 0.816 |  | 0.816 |
| 0 | 0.884 (0.666-1.174) | 0.396 | 0.889 (0.667-1.184) | 0.420 |
| <0.2 | 0.907 (0.673-1.222) | 0.520 | 0.911 (0.674-1.232) | 0.546 |
| 0.2≤duration<0.4 |  |  |  |  |
| ≥0.4 | 1.114 (0.459-2.701) | 0.812 | 1.158 (0.473-2.830) | 0.748 |

Model 1: unadjusted model

Model 2: multivariate regression model, if not stratified, adjusting for age, BMI, gender, marital status, education and vigorous activity

Supplementary Table 2 Associations between siesta, sleep duration at night, total sleep duration, the ratio of siesta in total sleep duration and CVDs in different subgroups

| Variable | Model 1 | | Model 2 | |
| --- | --- | --- | --- | --- |
|  | OR (95%CI) | *P* | OR (95%CI) | *P* |
| Male ≥60 years |  |  |  |  |
| Siesta duration (h) |  | 0.285 |  | 0.352 |
| 0 | 0.662 (0.402-1.090) | 0.105 | 0.743 (0.446-1.237) | 0.254 |
| <0.5 | 0.488 (0.107-2.220) | 0.353 | 0.461 (0.099-2.154) | 0.325 |
| 0.5≤duration<1 |  |  |  |  |
| 1≤duration<1.5 | 0.622 (0.366-1.054) | 0.078 | 0.662 (0.387-1.132) | 0.131 |
| ≥1.5 | 0.833 (0.500-1.387) | 0.482 | 0.918 (0.546-1.542) | 0.746 |
| Sleep duration at night (h) |  | 0.198 |  | 0.278 |
| <6 | 1.223 (0.882-1.694) | 0.227 | 1.218 (0.876-1.695) | 0.241 |
| 6≤duration<8 |  |  |  |  |
| ≥8 | 0.866 (0.597-1.258) | 0.451 | 0.900 (0.618-1.312) | 0.584 |
| Sleep duration (h) |  | 0.345 |  | 0.395 |
| <6 | 1.229 (0.847-1.784) | 0.277 | 1.249 (0.856-1.823) | 0.250 |
| 6≤duration<8 |  |  |  |  |
| ≥8 | 0.936 (0.675-1.300) | 0.694 | 0.975 (0.700-1.359) | 0.882 |
| Siesta ratio |  | 0.499 |  | 0.506 |
| 0 | 0.793 (0.547-1.149) | 0.220 | 0.803 (0.552-1.168) | 0.250 |
| <0.2 | 0.835 (0.578-1.206) | 0.337 | 0.787 (0.542-1.142) | 0.207 |
| 0.2≤duration<0.4 |  |  |  |  |
| ≥0.4 | 1.335 (0.488-3.657) | 0.574 | 1.197 (0.431-3.327) | 0.730 |
| Males <60 years |  |  |  |  |
| Siesta duration (h) |  | 0.112 |  | 0.137 |
| 0 | 1.151 (0.632-2.097) | 0.646 | 1.201 (0.656-2.200) | 0.553 |
| <0.5 | 2.203 (0.682-7.115) | 0.187 | 2.014 (0.615-6.596) | 0.247 |
| 0.5≤duration<1 |  |  |  |  |
| 1≤duration<1.5 | 1.701 (0.929-3.115) | 0.085 | 1.735 (0.944-3.192) | 0.076 |
| ≥1.5 | 1.683 (0.901-3.145) | 0.103 | 1.743 (0.925-3.285) | 0.086 |
| Sleep duration at night (h) |  | 0.322 |  | 0.339 |
| <6 | 1.317 (0.919-1.888) | 0.134 | 1.315 (0.913-1.893) | 0.142 |
| 6≤duration<8 |  |  |  |  |
| ≥8 | 1.087 (0.735-1.607) | 0.677 | 1.102 (0.742-1.636) | 0.630 |
| Sleep duration (h) |  | 0.534 |  | 0.500 |
| <6 | 1.050 (0.675-1.634) | 0.828 | 1.053 (0.674-1.645) | 0.819 |
| 6≤duration<8 |  |  |  |  |
| ≥8 | 1.209 (0.860-1.700) | 0.275 | 1.224 (0.867-1.728) | 0.251 |
| Siesta ratio |  | 0.228 |  | 0.261 |
| 0 | 0.687 (0.449-1.050) | 0.083 | 0.681 (0.444-1.044) | 0.078 |
| <0.2 | 0.925 (0.616-1.389) | 0.706 | 0.883 (0.585-1.333) | 0.553 |
| 0.2≤duration<0.4 |  |  |  |  |
| ≥0.4 | 0.443 (0.058-3.370) | 0.431 | 0.427 (0.056-3.273) | 0.413 |
| Females ≥60 years |  |  |  |  |
| Siesta duration (h) |  | 0.026 |  | 0.034 |
| 0 | 0.983 (0.620-1.557) | 0.941 | 0.965 (0.605-1.540) | 0.882 |
| <0.5 | 2.038 (0.862-4.821) | 0.105 | 1.964 (0.826-4.666) | 0.126 |
| 0.5≤duration<1 |  |  |  |  |
| 1≤duration<1.5 | 1.582 (0.973-2.572) | 0.065 | 1.545 (0.945-2.525) | 0.083 |
| ≥1.5 | 1.240 (0.733-2.099) | 0.423 | 1.225 (0.721-2.080) | 0.454 |
| Sleep duration at night (h) |  | 0.006 |  | 0.005 |
| <6 | 1.596 (1.176-2.166) | 0.003 | 1.617 (1.187-2.204) | 0.002 |
| 6≤duration<8 |  |  |  |  |
| ≥8 | 1.116 (0.766-1.625) | 0.568 | 1.124 (0.770-1.642) | 0.544 |
| Sleep duration (h) |  | 0.087 |  | 0.092 |
| <6 | 1.383 (1.005-1.902) | 0.046 | 1.388 (1.004-1.918) | 0.047 |
| 6≤duration<8 |  |  |  |  |
| ≥8 | 1.048 (0.747-1.470) | 0.786 | 1.053 (0.748-1.482) | 0.767 |
| Siesta ratio |  | 0.122 |  | 0.125 |
| 0 | 0.716 (0.500-1.024) | 0.067 | 0.720 (0.501-1.036) | 0.077 |
| <0.2 | 0.990 (0.683-1.434) | 0.956 | 1.006 (0.691-1.465) | 0.974 |
| 0.2≤duration<0.4 |  |  |  |  |
| ≥0.4 | 0.891 (0.326-2.434) | 0.821 | 0.913 (0.333-2.506) | 0.860 |
| Females <60 years |  |  |  |  |
| Siesta duration (h) |  | 0.001 |  | 0.001 |
| 0 | 1.416 (0.944-2.124) | 0.092 | 1.398 (0.930-2.102) | 0.107 |
| <0.5 | 3.093 (1.617-5.916) | 0.001 | 2.946 (1.529-5.673) | 0.001 |
| 0.5≤duration<1 |  |  |  |  |
| 1≤duration<1.5 | 0.968 (0.607-1.544) | 0.893 | 0.951 (0.595-1.520) | 0.834 |
| ≥1.5 | 1.066 (0.653-1.740) | 0.798 | 1.004 (0.613-1.645) | 0.987 |
| Sleep duration at night (h) |  | 0.001 |  | <0.001 |
| <6 | 1.315 (1.021-1.693) | 0.034 | 1.376 (1.065-1.778) | 0.015 |
| 6≤duration<8 |  |  |  |  |
| ≥8 | 0.701 (0.509-0.965) | 0.029 | 0.714 (0.518-0.985) | 0.040 |
| Sleep duration (h) |  | <0.001 |  | <0.001 |
| <6 | 1.221 (0.934-1.595) | 0.144 | 1.280 (0.977-1.678) | 0.074 |
| 6≤duration<8 |  |  |  |  |
| ≥8 | 0.599 (0.450-0.799) | <0.001 | 0.594 (0.445-0.793) | <0.001 |
| Siesta ratio |  | 0.185 |  | 0.139 |
| 0 | 1.350 (0.942-1.935) | 0.102 | 1.400 (0.973-2.012) | 0.070 |
| <0.2 | 1.068 (0.727-1.569) | 0.736 | 1.095 (0.743-1.613) | 0.646 |
| 0.2≤duration<0.4 |  |  |  |  |
| ≥0.4 | 0.983 (0.287-3.371) | 0.978 | 1.043 (0.302-3.608) | 0.947 |

Model 1: unadjusted model

Model 2: multivariate regression model, if not stratified, adjusting for age, BMI, gender, registered permanent residence, education and vigorous activity
